# Titin Is Present in the Elastic Tethers That Connect Separating Anaphase Chromosomes in Crane‐Fly Spermatocytes

**DOI:** 10.1002/cm.70035

**Published:** 2025-09-09

**Authors:** Demetra Economopoulos, Maral Janan, Martina Krüger, Aavo‐Valdur Mikelsaar, Arthur Forer, Rose Sheykhani

**Affiliations:** ^1^ Department of Biology York University Toronto Canada; ^2^ Institute of Cardiovascular Physiology, Medical Faculty and University Hospital Düsseldorf, Heinrich Heine University Düsseldorf Düsseldorf Germany; ^3^ The Institute of Biomedicine and Translational Medicine, Faculty of Medicine University of Tartu Tartu Estonia; ^4^ Department of Biology Lakeland Community College Kirtland Ohio USA

**Keywords:** chromosomes, connectin (titin), fluorescent antibody technique, meiosis, mitotic tethers, myofibrilsanaphase

## Abstract

Elastic tethers connect telomeres of separating chromosomes in anaphase of animal cells. Immunofluorescence staining of titin in crane‐fly spermatocytes, using 4 different antibodies, shows that the giant elastic protein titin seems to be a component of mitotic tethers: titin “strands” extend between separating chromosomes, connecting their telomeres, just as tethers do. Since titin is responsible for elastic forces in myofibrils, we suggest that titin is responsible for the backwards forces exerted on chromosome arms during anaphase.

## 1 Introduction

We show in this article that the giant elastic protein “titin” is a component of the mitotic spindle and, in particular, we propose that it is a component of elastic *tethers* extending between telomeres of separating anaphase chromosomes. Mitotic tethers are elastic physical connections between telomeres of separating anaphase chromosomes that previously were paired. Evidence that there are physical connections between anaphase chromosomes (in crane‐fly spermatocytes) was presented by LaFountain et al. (2002). They cut pieces from chromosome arms (with a laser microbeam) during anaphase, and the resultant arm fragments rapidly moved backward toward the chromosome moving to the opposite pole, telomere moving to telomere. The backward movements required both telomeres because ablation of either telomere stopped arm fragment movement (LaFountain et al. 2002; Forer et al. 2021). LaFountain et al. (2002) concluded that tethers physically connect separating anaphase chromosomes. The movements of chromosome arm fragments are not due to microtubules because arm fragments do not move when their telomeres are ablated, and they move at their usual speed when taxol very much slows down anaphase chromosome movement (Forer et al. 2018). Arm fragment movements are not due to ultrafine DNA‐bridges because the mitotic tethers are associated with each chromosome, whereas DNA‐bridges are mostly between centromeres, and those between telomeres are present in far fewer numbers than one per chromosome pair: more than 20% of cells have no such bridges (Gemble et al. 2015, 2016; Nielsen et al. 2015; Kong et al. 2023). Another kind of anaphase DNA‐containing bridges was described by Warecki et al. (2023) in *Drosophila* neuroblasts, identified as containing DNA by staining with DAPI. These are not the same as the mitotic tethers because tethers are present in all cells, associated with all chromosomes, whereas the Warecki et al. (2023) bridges were seen in less than 40% of the cells, and only one per cell was described. In addition, there may be questions about those data because the neuroblasts were not kept in isotonic or balanced media: they were placed in PBS, which contains 0.84% NaCl and is isotonic to mammalian cells, or they were placed in non‐buffered 0.7% NaCl, which is not isotonic to insect tissue and is missing buffer and other salts. In sum, the mitotic tethers identified by severing chromosome arms are different from microtubules and from the DNA‐containing bridges between anaphase chromosomes described elsewhere.

As further evidence that tethers are different from microtubules and the other bridges, electron microscope tomograms have illustrated mitotic “tethers” in crane‐fly spermatocytes: the structures physically link separating telomeres of partner chromosomes, are present in the numbers expected, and they appear as ~40 nm diameter bipartite structures that connect separating telomeres, with substructural components that appear as ~5 nm diameter filaments (Forer and Otsuka 2023).

Evidence for mitotic tethers is present in other cell types as well as crane‐fly spermatocytes, as identified by backward movement of arm fragments. In addition to primary and secondary spermatocytes of crane flies (LaFountain et al. 2002), tethers connect chromosome arms in a phylogenetically broad range of animal mitotic and meiotic cells: in primary spermatocytes of an aquatic Turbellarian flatworm (*Mesostoma*), in primary and secondary spermatocytes of the house cricket *Acheta* (an Orthopteran insect), in primary spermatocytes of cellar spiders (*Pholcus*) and black widow spiders (*Latrodectus*), in mitotic marsupial (PtK) cells, and in mitotic human (U2OS) cells (Forer et al. 2017). Thus, mitotic tethers are phylogenetically widespread and perhaps may be a conserved feature of chromosomes in animal cells. Tethers seem to connect each separating pair of anaphase chromosomes, based on the very high frequency of chromosome arm fragments that move. However, not all arms of individual chromosomes are connected by tethers to their partner chromosomes. In crane‐fly primary spermatocytes only two of the four arms are connected to the partner chromosome (LaFountain et al. 2002; Sheykhani et al. 2017), and in PtK cells only one of the two arms is connected to the partner chromosome (Forer et al. 2017), the only two cell types tested for this.

Mitotic tethers lose elasticity during anaphase. Chromosome arm fragments formed with short tethers (i.e., with small distance between separating telomeres) almost invariably move completely to the partner telomere. Arm fragments formed with medium length tethers move part way to their partners. And arm fragments formed with longer tethers (in late anaphase) do not move at all. These conclusions hold for both crane‐fly spermatocytes (LaFountain et al. 2002; Forer et al. 2021) and PtK cells (Forer et al. 2017), the only cells tested in this regard. The tethers remain attached to the two separated telomeres in late anaphase and into telophase, but they are not elastic (Forer et al. 2017). We know they are still attached because when tethers are cut directly, the two connected arms shorten: they contract by about 10% of their original length. Similarly, when an arm fragment is formed by severing an arm, the non‐amputated arm contracts by about 10%. The arms contact by the same amount at all tether lengths—i.e., even at tether lengths at which the arm fragments do not move when produced at that tether length. Thus, tethers are still in place during late anaphase and inelastic tethers put the same tension on the chromosome arms as do elastic tethers.

Current evidence suggests that loss of tether elasticity (in crane‐fly spermatocytes) is because of dephosphorylation of tethers, which starts after the telomeres have separated by ~3–5 μm (Kite and Forer 2020; Forer et al. 2021). Fabian, Troscianczuk, and Forer (2007) observed that when CalyculinA, an inhibitor of PP1, is added to cells in early anaphase, the chromosomes move backward after reaching the poles, telomeres moving to sister telomeres, dragging the kinetochores behind them. Thus, blocking PP1 with CalA preserves tether elasticity through anaphase. [CalyculinA blocks both PP1 and PP2A at the concentrations of Calyculin A used to treat the cells, but various control experiments showed that PP1 is the component that dephosphorylates the tethers, not PP2A (Kite and Forer 2020).] The backward movement caused by Calyculin A was shown to be due to tethers by laser cutting and laser ablation experiments (Forer et al. 2021).

**FIGURE 1:**
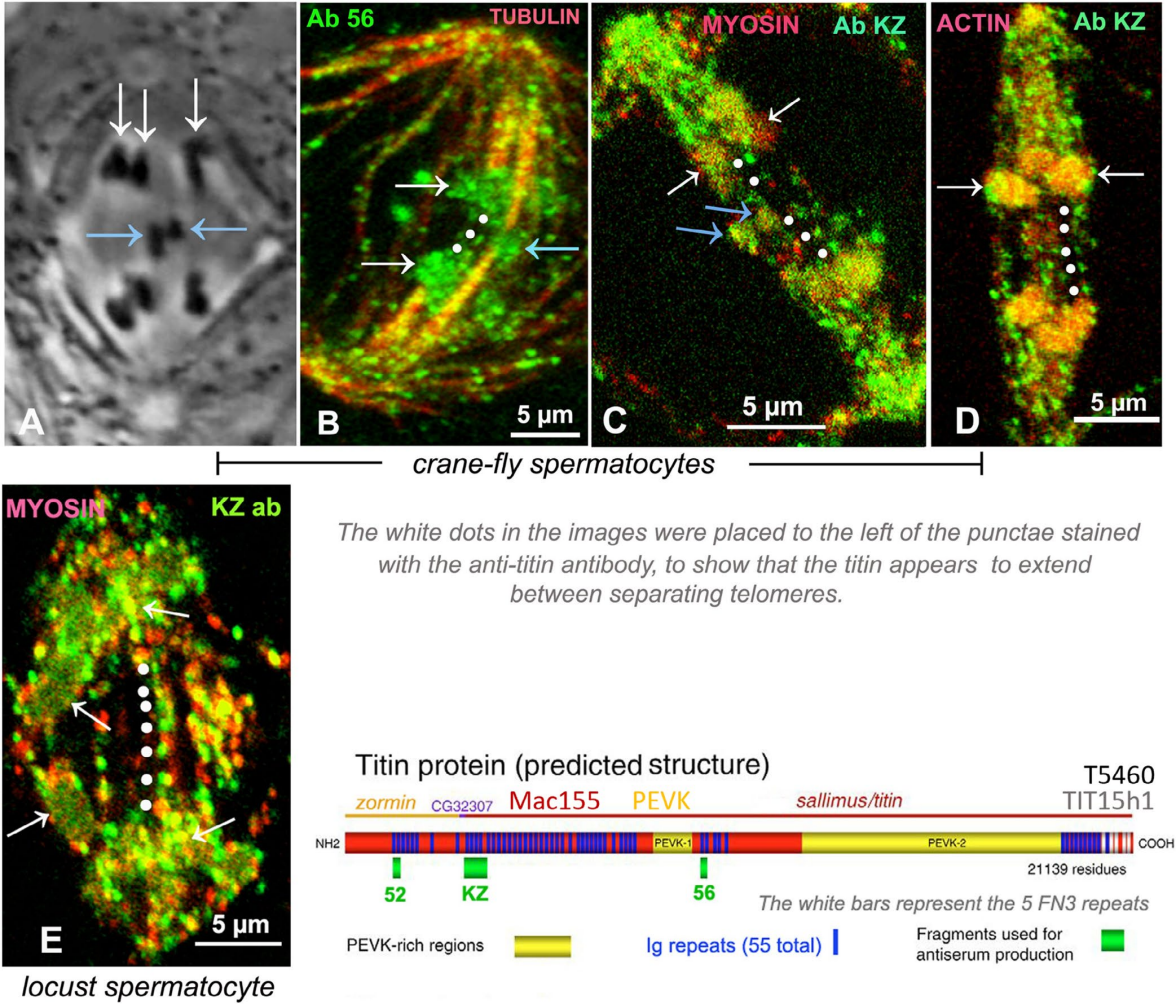
Shows images of crane‐fly spermatocytes and an image of a locust spermatocyte. (A) Is a phase‐contrast microscopy image of a living anaphase crane‐fly spermatocyte showing all three pairs of separating half‐bivalents (dyads). White arrows point to half‐bivalents moving to the top pole. The three pairs of half‐bivalents are approaching the two poles while the 2 sex chromosomes (blue arrows) still are at the equator: In these cells the sex chromosomes do not move to the poles until after the autosomes reach the poles (B–D) Are confocal microscope images of crane‐fly spermatocytes stained with antibodies αKZ or α56, colourised green, as indicated by the label on each image, together with a second stain of Tubulin, Actin or Myosin which appear red, as indicated by the label on each image. (E) Is of a locust spermatocyte. The fluorescently stained images were originally published in Fabian, Xia, et al. (2007); we modified them for this figure. The antibodies were derived from *Drosophila* titin immunogens, the positions of which are indicated in the drawing (which was modified from a drawing in Fabian, Xia, et al. 2007). A third antibody (not shown in a fluorescence image), α52, gave the same staining pattern. The positions of the current study's antibodies are approximated and annotated based on epitope information from published sources (see Methods), and are mapped here to facilitate comparison with previous studies. In (B–E) we added solid white circles to the left of punctate staining by anti‐titin antibodies to point to the apparent continuity of titin between the two separating telomeres. The white arrows point to separating half‐bivalents, and in (A–C) the blue arrows point to the sex chromosomes at the equator.

What are tethers composed of? We have no data on what tethers are composed of. Fabian, Troscianczuk, and Forer (2007); Fabian, Xia, et al. (2007) suggested that tethers contain the giant protein titin, the major elastic component in skeletal and cardiac muscle and the third most abundant protein in myofibrils (e.g., Linke et al. 1998; Tskhovrebova and Trinick 2003; Krüger and Linke 2006; Hamdani et al. 2017). Fabian, Troscianczuk, and Forer (2007); Fabian, Xia, et al. (2007) suggested this because they had found titin immunofluorescence between separating chromosome arms in both locust and crane‐fly spermatocytes (Fabian, Troscianczuk, and Forer 2007; Fabian, Xia, et al. 2007). Their immunofluorescence staining was with three different antibodies that labeled three different non‐repetitive regions of the titin gene product in *Drosophila*: each of the three antibodies stained punctae that were arranged linearly between sister telomeres in anaphase (Fabian, Troscianczuk, and Forer 2007; Fabian, Xia, et al. 2007). We have reproduced some of their images (from Fabian, Xia, et al. 2007) and present them in Figure 1. Since titin was present between separating anaphase chromosomes and seemed to connect separating telomeres, they suggested that titin might be a component of mitotic tethers.

If tethers contain titin, titin must be present in one continuous strand that connects telomeres of separating anaphase chromosomes. The titin staining from Fabian, Troscianczuk, and Forer (2007); Fabian, Xia, et al. (2007) is convincing of the *presence* of titin between separating chromosomes, but that the arms are physically *connected* by titin is not as convincing: it is possible that the linearity of the punctate staining does not necessarily mean that the punctae are physically connected to each other or to the telomeres they seem to run between. To deal with whether titin connects separating telomeres, we studied titin again in crane‐fly spermatocytes, one of the cell types studied by Fabian, Xia, et al. (2007), using four other different anti‐titin antibodies to test if immunofluorescence with these antibodies supports the possibility that linear titin strands connect separating telomeres. In this article, we show that titin strands extend between and connect the arms of separating partner chromosomes in anaphase, physically connecting them. Importantly, we also show that the tethers in primary and secondary spermatocytes match the numbers and distributions expected from the laser‐microbeam arm‐fragment experiments, i.e., in *primary spermatocytes* partner chromosomes are connected by no more than two tethers in any given separating chromosome pair, there are no connections between telomeres that are not partners, and in *secondary spermatocytes* no more than one tether connects arms of partner chromosomes in any given separating pair. We also show that all four antibodies stain other regions of the spindle as well as the mitotic tethers, suggesting that titin is involved in general spindle functions and not just as mitotic tethers.

## 2 Materials and Methods

### 2.1 Preparing Living Cells

Our experiments were on spermatocytes of the crane fly *Nephrotoma suturalis* (Loew). We reared crane flies in the laboratory using techniques basically as described in Forer (1982). Spermatocytes in individual crane fly testes all enter metaphase‐I within an approximately 2‐day period during the 4th larval instar. We obtained dividing spermatocytes using procedures described in detail in Forer and Pickett‐Heaps (2005). Briefly, fourth‐instar crane‐fly larvae with testes in the appropriate stage were covered with Halocarbon oil (Sigma) and the testes were removed and placed in a small drop of Halocarbon oil. The oil was rinsed off in Ephrussi‐Beadle *Drosophila* Ringers solution (Ephrussi and Beadle 1936) to which we added phosphate buffer. The Ringers solution contained final concentrations of 0.13 M NaCl, 5 mM KCl, 1 mM CaCl_2_, and 3–5 mM phosphate buffer, pH 6.8–6.9. After rinsing the oil away, individual testes were placed on a previously flamed coverslip in a drop of Ringers solution which contained fibrinogen. Once the testes were broken open and the cells spread around in the drop, thrombin was added to form a clot and the coverslip was then inverted over Ringers solution in a perfusion chamber (described in detail in Forer and Pickett‐Heaps 2005).

### 2.2 Preparing Living Cells for Immunofluorescence

We used phase‐contrast microscopy to determine that there were anaphase cells in the preparation, after which the coverslip was immersed in lysis buffer, a cytoskeleton‐stabilizing buffer that contains DMSO and detergent, of composition: 100 mM Pipes buffer, final pH 6.8–6.9; 10 mM EGTA; 5 mM MgSO4; 5% DMSO; and 1% Nonidet P‐40 or its substitute, Igepal. The rationale for using this method is that by lysing the cell and only studying the bare cytoskeleton, the only components in the preparation are cytoskeletal: all soluble material is washed out of the cell as well as any other material not stabilized with the cytoskeleton. This greatly reduces the chances that the antibodies will label off‐target components: anything that is labeled is part of the cytoskeleton. As an indication that lysis before fixation does not introduce large morphological changes, this method for preparing cells for immunostaining preserves the shape of spindles and positions of anaphase chromosomes and preserves the positions of severed kinetochore microtubules produced by microbeam irradiations (e.g., illustrations in Forer et al. 2003; Sheykhani, Baker, et al. 2013; Forer et al. 2018).

After 10–15 min or more in the lysis solution, the cells were fixed for 5 min with 0.25% glutaraldehyde (in insect Ringers solution), rinsed several times by immersion in PBS [Phosphate Buffered Saline—pH 6.9], and then, to neutralize free aldehyde groups, they were placed for 10–15 min in PBS that contained 0.05 M glycine. After rinsing several times in PBS, the coverslips were immersed in a 1:1 mixture of glycerol:PBS and stored at 4°C until used for immunostaining.

### 2.3 Preparing Glycerinated Myofibrils

We studied stained myofibrils to test that the antibodies bound to titin, and to see which part of the extended titin molecule the antibodies bound to. We did not study muscles fixed in situ but rather studied the isolated contractile muscle proteins kept in place when the myofibrils are glycerinated and removed from the muscle. There were no other molecules in the myofibrils other than the contractile cytoskeleton. The procedure for obtaining myofibrils from adult crane‐fly thoraces is described in detail in Fabian, Xia, et al. (2007). Briefly, we decapitated living crane flies, removed the abdomen from the body, and then removed muscles from the thorax. During dissection of the muscles, the thorax was kept in muscle standard salt solution [SSS], composed of 50 mM KCl, 1 mM MgCl_2_ and 5 mM phosphate buffer, pH 6.9. Once the thorax was removed, the muscle was placed in a 1:1 mixture of glycerol: SSS, vigorously vortexed, and stored at −20°C for days to months to years until used for immunostaining, at which time they were present as single myofibrils or several joined myofibrils, isolated from membranes or other cell proteins, myofibrils with no other muscle protein than the cytoskeleton but that were still contractile upon addition of ATP. Rabbit myofibrils were obtained similarly from glycerinated psoas muscles that were macerated after immersion in glycerol.

### 2.4 Preparing Myofibrils for Immunostaining

To attach the myofibrils to coverslips for immunostaining, an aliquot of glycerinated myofibrils in 1:1 glycerol/Standard‐Salt‐Solution was suspended in SSS, spun down in a table‐top centrifuge, resuspended in SSS, and spun down again. The pellet was suspended in a small amount of SSS; for each coverslip preparation, 3 μL of this myofibril solution was mixed with 5 μL fibrinogen (in SSS), then 5 μL thrombin (in SSS) was added. Once the clot formed, the myofibrils were fixed using 0.25% glutaraldehyde (in SSS or in insect Ringers solution) and then treated as described above for treatment of spermatocytes: the coverslip preparations were rinsed, incubated with glycine, rinsed, and stored at 4°C in a 1:1 mixture of glycerol:SSS.

### 2.5 Immunostaining

The entire immunostaining procedure was at room temperature. Glycerol was removed from the coverslips by floating them cell‐side down on PBS (so that the heavier glycerol sinks) for varying times from 10 to 60 min. The coverslips were then rinsed by immersion in PBS, and prior to incubating with primary antibodies, were rinsed by squirting the coverslip with PBS that contained 0.05% Triton‐X (to facilitate spreading of the antibody). They then were incubated with primary antibodies for an hour, rinsed twice with PBS, squirted with PBS/Triton, incubated with secondary antibodies for an hour, then rinsed as before. For those preparations stained with phalloidin, phalloidin was added after the primary and secondary antibodies were rinsed after treating with the secondary antibody. An aliquot of a 6.6 μM stock of 488‐Alexa phalloidin in EtOH (invitrogen) was evaporated overnight in a fume hood and resuspended in PBS at the final concentration used for staining, 0.05–0.1 μM for different runs. After rinsing with PBS, each coverslip finally was placed on a slide onto a drop of Mowiol‐containing solution (Osborn and Weber 1982) to which we added 0.04% para‐phenylene‐diamine as an anti‐fading agent. The slides were left in the fume hood (in the dark) for the Mowiol to harden. Once the Mowiol hardened (which took overnight or a day) we studied the preparations using confocal microscopy.

### 2.6 Primary Antibodies

We used four different antibodies to stain for titin.

1. Tit1 5H1.1 is a mouse monoclonal antibody (IgG1) obtained from Aavo‐Valdo Mikelsaar. Mikelsaar et al. (2010) obtained the antibody using as immunogen a 19 amino acid synthetic polypeptide found in the A‐band region of human titin, corresponding to the C‐terminal part of fibronectin type‐III domain 103. They later deduced that the antibody responds to a 6 amino acid sequence, AVNKYG (Mikelsaar et al. 2012), a sequence found in several fibronectin domains. BLAST analysis indicates that a four amino acid sequence (NKYG) within that 6‐amino‐acid sequence is found in 13 isoforms of *Drosophila melanogaster* titin and that the same four amino acid sequence and up to all 6 amino acids are found in titin in other multiple titin isoforms in other *Drosophila* species, including *Drosophila miranda, obscura, pseudoobscura, subobscura, willistoni, mohavensis, persimilis, guanche*, and *navojoa*, and in mosquito species (e.g., *Culex quinquefasciatus* and *Culex pipiens pallens*), all close relatives to crane flies.
2. T5460 is a polyclonal rabbit antibody (IgG) obtained from Kathy Wilson (Zastrow et al. 2006). The immunogen for T5460 is residues 61–172 from the C‐terminus of human titin in domain M‐is6 in Titin 1–551, the C‐terminal 551 residues at the C‐terminus (Zastrow et al. 2006). Titin region 1–551 is located in the M‐band of human skeletal muscle.
3. PEVK is a polyclonal rabbit antibody (IgG), generated by Immunoglobe (Würzburg, Germany) for the peptide sequence CEVVLKSVLRKR in the PEVK region (titin peptide sequence UniProtKB: Q8WZ42) and was obtained from Martina Krüger. Antibody specificity was tested using recombinant PEVK peptide as previously reported (Kötter et al. 2013). The titin PEVK region is located in the I‐band of mammalian cardiac and skeletal myocytes. BLAST analysis indicates that the sequences EVVLK, EVVLKS, and up to EVVLKSVLR are found in titin isoforms in various *Drosophila* species (e.g., *melanogaster, rhopaloa, santomea, yakula*, and *tiessieri*) as well as in various Culicinae mosquitoes (e.g., *Culex quinquefasciatus, Culex pipiens pallens*, *Malaya genurostris, Topomyia yanbarensis, Aedes albopictus*), all close relatives to crane flies.
4. KETTIN antibody [MAC 155] is a rat IgG1 monoclonal antibody purchased from Abcam [ab50585, Abcam Inc., Toronto, www.abcam.com]; this antibody no longer is available from Abcam, but it is available at Developmental Studies Hybridoma Bank (DSHB) at the University of Iowa. According to the Abcam data sheet, the immunogen consisted of Z‐discs purified from *Lethocerus indicus* (a waterbug) The antibody reacts with 500 and 700 kDa isoforms in *Lethocerus* and *Drosophila* muscle, both flight and non‐flight muscle, near the middle of the molecule. The data sheet noted that the antibody also reacts with *C. elegans* muscle, but not with rabbit muscle. In other references to what seems to be the same antibody (though not explicitly stated that this is the same antibody sold by Abcam), antibody “MAC 155” is described as a rat IgG1 monoclonal antibody obtained using Z‐discs from *Lethocerus indicus* as immunogen (Lakey et al. 1990, 1993), purified from MAC 155 hybridoma cell supernatant (Lakey et al. 1993), and referred to as MAC‐155 antibody. MAC‐155 binds to kettin, a titin homolog in insects (Burkart et al. 2007). Specifically, kettin is the most abundant isoform of sallimus in adult flies, one of two shorter proteins that represent the function of vertebrate titin. Fragments of kettin protein to which MAC‐155 binds also bind to actin and actinin, but do not bind (or binds much less) to myosin (Lakey et al. 1990). The antibody itself binds to the recombinant linker‐Ig‐linker fragment of *Drosophila* kettin, which includes Klg16 (Burkart et al. 2007).

### 2.7 Secondary Antibodies, and Confocal Microscopes

Different preparations were studied using one of various confocal microscopes: either an Olympus Fluoview confocal microscope, a Zeiss LSM confocal microscope, or a Nikon confocal microscope, using each microscope's 60× objective lens, NA = 1.4. The results were interchangeable. *Myofibrils* were stained for actin using Phalloidin Alexa Fluor 488, and for titin with a secondary antibody with a fluorophore appropriate for the laser line near 560 nm (depending on the microscope), namely Alexa 568 or DyLight 550 and, for the Olympus microscope, for the laser line at 633 nm (DyLight 650, or Alexa Fluor 633). *Spermatocytes* were stained for titin using secondary antibodies Alexa 488, or Alexa 568, or Alexa 633, or DyLight 550 or DyLight 650. The Olympus Fluoview was the only microscope for which we stained preparations using fluorophores in the far red (> 600 nm).

We originally stained one primary antibody at a time—e.g., first adding primary mouse antibody, then secondary antibody against mouse IgG, then primary rat antibody, then secondary antibody against rat IgG. But because we purchased only secondary antibodies that were highly cross‐adsorbed (by the supplier) against antibodies outside the target primary antibodies, all primary antibodies later were added simultaneously followed by all secondary antibodies added simultaneously. We did not detect any cross‐staining. The stock primary antibodies were used after dilution with PBS to the following concentrations: Tit1 5H1.1 from 1 to 250 or 1 to 500. T5460 from 1 to 500. PEVK from 1 to 500. MAC‐155(kettin) from 1 to 200 or 1 to 400.

All secondary antibodies were used as stock solutions diluted 1:100 or 1:200 with PBS. Confocal images were analyzed using Fiji‐Image J (e.g., to study the Z‐stacks of images, to obtain magnifications, to sum specified images in the Z‐stacks) and were put into montages using Photoshop (e.g., to trim and place images in montages, to label the montages, etc.).

In presenting the confocal microscopy results, we used Fiji/Image J to project and intensity‐sum various numbers of Z‐slices to form a composite image. The number of slices we used for the different images is presented under the images in question (e.g., Figure 3).

## 3 Results

### 3.1 Glycerinated Crane‐Fly Myofibrils

We stained glycerinated crane‐fly myofibrils to test whether the antibodies bound to titin in crane‐fly skeletal muscle and to determine which part(s) of the titin they bound to. [Titin molecules in skeletal muscle exist as single linear strands over 1 μm long, extending in each sarcomere from the middle of the A‐band to a Z‐line, essentially connecting the two Z‐lines that define a sarcomere; consequently, the positions along the titin molecules that react with the antibody can be determined by the staining pattern in the myofibril.]

**FIGURE 2:**
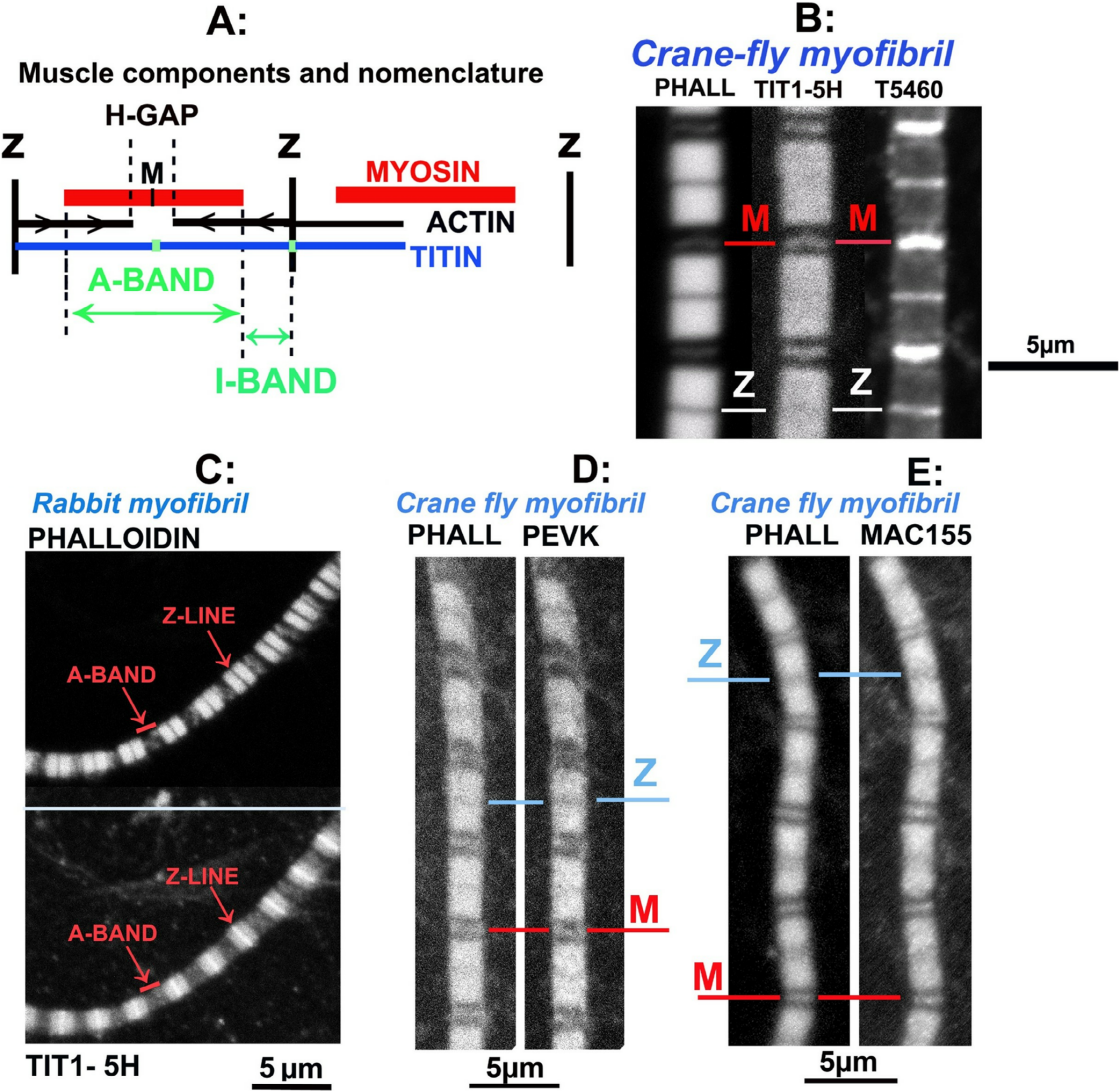
(A) Is a schematic illustration of myofibril components and the nomenclature used in our descriptions. The A‐band is synonymous with the myosin location. The I‐band (when present) is the less phase‐dense space between myosin and the Z‐line. The H‐gap when present is the less phase‐dense area in the A‐band. (We studied primarily myofibrils with clear Hgaps.) The M‐band is I the middle of the A‐band. Titin molecules extend from the M‐band to the Z‐line, and their termini are indicated in green. The arrowheads on the individual Actin filaments represent the filament polarities. The remaining images (A–E) are fluorescently stained images of glycerinated myofibrils. Those in (B), (D) and (E) are from crane‐fly thoracic muscles, and those in (C) are from rabbit psoas muscles. The same myofibril is presented in each triplet or doublet image, the images lined up to help in comparing staining in the same sarcomeres. Each myofibril is stained with phalloidin, which stains only Actin, and with 1 or 2 of the anti‐titin antibodies, T5460, Tit1‐5H1.1, PEVK or MAC155 (kettin) as indicated on the label next to the image. The labels on the images point to the M‐band (labeled M), the Z‐line (labeled Z) separating the Actin filaments in two adjacent sarcomeres, and the middle of the A‐band, where there are no Actin filaments. In these and subsequent images the magnifications are indicated by labeled scale bars.

We stained glycerinated *crane‐fly myofibrils* both with fluorescent phalloidin and with one or two of the four anti‐titin antibodies we used to study spindles. The phalloidin binds solely to actin and allows us to delineate the structure of the myofibrils in question and hence which part of the muscle reacts with that antibody. The overall conclusion is that each of the 4 antibodies stained crane‐fly myofibrils, with different of the antibodies having different or similar staining patterns along the length of the myofibrils, as illustrated in Figure 2B–E; for those who may not be familiar with the arrangements of components in myofibrils, or with the nomenclature used to describe positions along sarcomeres, we include a cartoon to illustrate the components and the nomenclature (Figure 2A).

We also stained glycerinated *rabbit* myofibrils with Tit1‐5‐H1.1. This is because Tit1‐5‐H1.1 was reported to specifically stain the A‐band in human, mouse, and zebrafish muscle (Mikelsaar et al. 2012) whereas in crane‐fly myofibrils, Tit1‐5‐H1.1 did *not* stain the A‐band except for the small M‐band in the middle of the A‐band. Rather, it consistently stained titin in the I‐band along the length of the actin filaments. To test whether the different pattern of staining in crane‐fly myofibrils was due to off‐target staining, we studied rabbit myofibrils; we stained both crane‐fly glycerinated thoracic myofibrils and rabbit glycerinated psoas muscle myofibrils on the same day, with the same solutions. The crane‐fly myofibrils were stained along the actin filaments and in the M‐band, as in Figure 2A. The rabbit myofibrils stained in the same pattern, as illustrated in Figure 2B. To further test whether there might be off‐target staining with Tit1‐5‐H1.1, we incubated the antibody overnight in PBS with small peptides [from Thermo‐Fisher, Toronto] that had the specific 6‐amino‐acid sequence that the antibody recognizes (AVNKYG), as described in Mikelsaar et al. (2012). The peptides blocked the staining along the actin filaments in both crane‐fly myofibrils and rabbit myofibrils, confirming that the staining is of the specific immunogen and that the different pattern of staining in crane‐fly myofibrils is not a result of antibody binding to a peptide sequence other than the target AVNKYG. Furthermore, BLAST analysis suggests that the target peptide sequence is not present in spindle microtubules, strengthening the interpretation of Tit1‐5‐H1.1 staining as reflective of the presence of titin within the spindle. While it is possible that the antibody may interact with proteins other than titin, it seems unlikely that these off‐target stains would overlap with microtubules and our proposed tethers.

### 3.2 Crane‐Fly Meiotic Spindles

Crane‐fly primary spermatocytes have 2*n* = 8 chromosomes; in pre‐anaphase spermatocytes, they appear as 3 autosomal bivalents plus two sex‐chromosome univalents. The chromosomes align at the equator by metaphase; at anaphase, the autosomal bivalents disjoin into half‐bivalents, which move to the poles while the sex chromosome univalents remain at the equator (Figure 1A). Each univalent has a kinetochore spindle fiber (containing a bundle of microtubules) to each pole. The sex chromosomes segregate to opposite poles only after the autosomes near or reach the poles.

Various spindle components in primary spermatocytes were stained by the three anti‐titin antibodies used by Fabian, Xia, et al. (2007). As well as staining mitotic tethers, their anti‐titin antibodies stained the nuclear membrane before prometaphase; stained chromosomes from prometaphase through telophase; stained spindles in general, including strong staining along kinetochore fibers and less strong staining in other microtubule‐containing areas of the spindle; stained the spindle poles; and in late anaphase stained cleavage furrows, reformed nuclei, and mid‐body microtubule regions. Our observations with the 4 different anti‐titin antibodies we used by and large agree with theirs. In this section, we highlight these findings and outline our hypothesis that some of the titin staining in the spindle is, in fact, representative of tethers.

#### 3.2.1 Titin Staining Throughout Spindles

**FIGURE 3:**
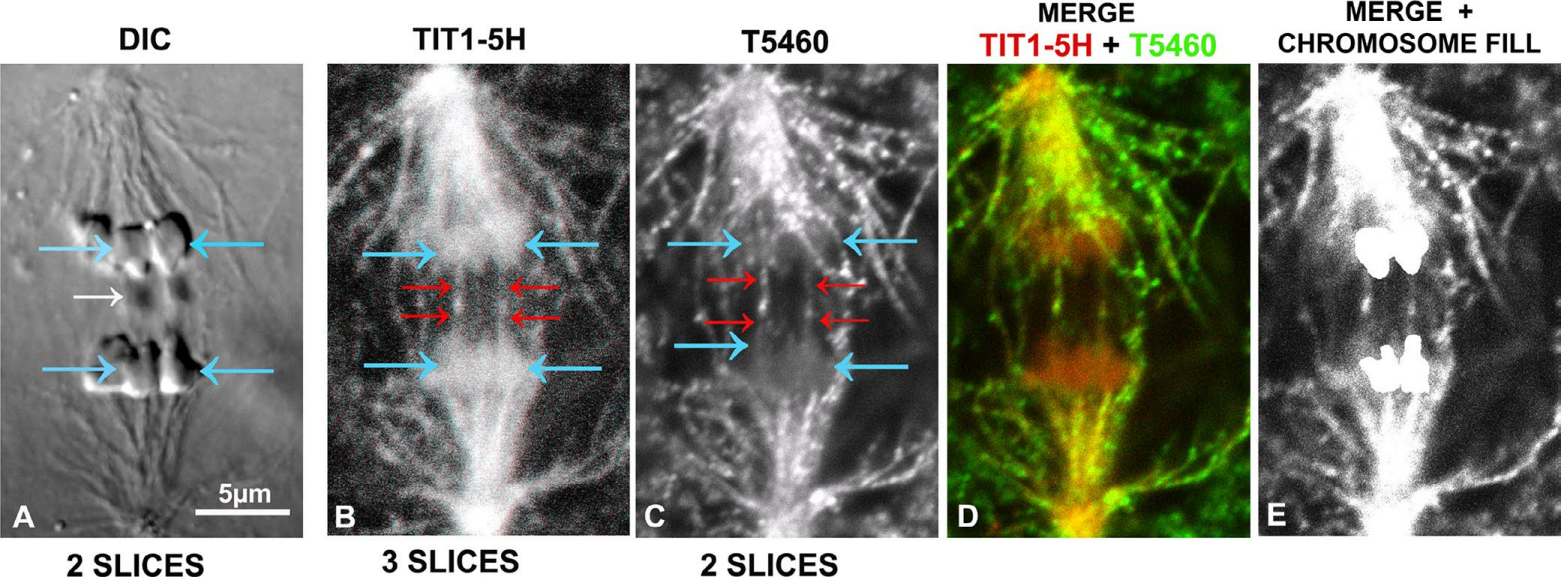
Shows a single cell double‐stained with anti‐titin antibodies Tit1‐5H1.1 and T5460, as labeled, together with a DIC image and a colourised and merged image (D) that illustrates that the titin strands identified in the single images (B, C) stain the same component titin strands. In (D) the Tit1‐5H1.1 image was colored red and the T5640 colored green. In this (and subsequent montages) the number of slices from the Z‐stack that were projected are listed under the images in question. The white arrow points to an out‐of‐focus sex chromosome. The blue arrows point to the chromosomes that are linked by the titin strands labeled with red arrows. (D) Is a stereo image (red‐green glasses) of the slices near the tethers. (E) Is a merged image with the positions of chromosomes pointed to in (A–C) filled in white, so that their positions are easier to distinguish. Tit1‐5H also strongly stained the spindle poles, the autosomes, the autosomal kinetochore fibers, and the spindle in general; T5460 stained the same components but stained chromosomes and poles less strongly than did TIT1‐5H.

Multiple regions of the spindle were stained with all four of the anti‐titin antibodies. Some stained regions contained microtubules. All four antibodies consistently strongly stained kinetochore fibers (Figures 1, 3, 5, 7, 8, 9, 11), as previously described by Fabian, Troscianczuk, and Forer (2007); Fabian, Xia, et al. (2007). The staining was strong, but often uneven (e.g., Figure 9). In other parts of the spindle, the staining sometimes appeared as linear strands, sometimes not; the staining was irregularly positioned in the body of the spindle and presumably was near (or associated with) some of the non‐kinetochore microtubules. Staining of the general spindle was weaker in intensity than staining of kinetochore spindle fibers that contain microtubule bundles (e.g., Figures 3, 6, 7), and sometimes spindle staining was somewhat punctate and uneven. Whereas staining with the titin antibodies allows one to discern a spindle shape, only sometimes is there staining of discrete identifiable microtubules, except for kinetochore fibers (which contain microtubule bundles). Notably, all four antibodies also stained chromosomes, as described by Fabian, Xia, et al. (2007) for the three anti‐titin antibodies they used (Figure 1). In our experiments, chromosomes stained intensely from metaphase through late anaphase except that antibody T5460 generally stained the chromosomes the weakest of the four antibodies (e.g., Figure 3B vs. C, Figure 11B vs. C). In later stages (mid‐anaphase through cleavage) titin antibodies stained the spindle poles strongly (Figures 3 and 7, 8, 9, 10, 11), stained long strands along the length (pole‐to‐pole) of dividing cells (Figures 8, 9 and 11), stained the cell cortex, and stained early cleavage furrows [Figure 11]. Thus, all four antibodies stained different parts of the spindle (especially kinetochore fibers and spindle poles); all stained the cell cortex, cleavage furrows, and chromosomes. The broad spindle staining observed across different anti‐titin antibodies suggests multiple roles for titin in cell morphology and division. These results lend support to our hypothesis that titin is also a component of tethers extending between the telomeres of separating half‐bivalents in anaphase.

#### 3.2.2 Inter‐Telomere Titin Staining (Tethers)

We consistently saw *titin strands* extending between the telomeres of separating anaphase chromosomes with each of the 4 antibodies [Tit‐1‐5‐H1.1; T5460; PEVK; MAC‐155]. We studied over 100 spermatocytes in anaphase, and we studied many cells in metaphase. In early and mid‐anaphase primary spermatocytes we generally saw at least one titin strand extending from a chromosome toward the partner telomere; we often could not follow them to the partner because they were obscured by sex chromosomes, or by stained sex‐chromosomal kinetochore fibers (in the interzone) or because the putative tether was at an angle to the plane of focus and hard to follow, even with confocal microscopy. We never saw titin strands extend toward (or contact) non‐partner chromosomes; they extended only toward their partner chromosomes. We were able to see clear titin strands best when they were considerably separated laterally or vertically from the interzonal sex‐chromosome kinetochore fibers and separated from nearby autosome arms in different but nearby planes of focus, or when their positions were such that there was no confusion with autosomal or sex‐chromosomal kinetochore fibers (all of which were stained by the antibodies in question). In some cells, we were able to identify titin strands connecting only one set of separating chromosomes; in some cells, we could see titin strands connecting several pairs of partners or even all pairs; but we never saw more than two connections per separating pair, and we never saw strands extending to non‐partner chromosomes. The titin strands between telomeres were especially clear when the cells were flat such that the separating chromosome pairs were in a plane of focus parallel to or close to parallel to the coverslip. We now illustrate and describe titin strands as seen with the different antibodies.

Titin strands were seen in cells double stained with Tit1‐5‐H1.1 and T5460, as illustrated in the early anaphase cell shown in Figure 3. Two separating pairs of chromosomes (blue arrows) were connected by titin strands (red arrows), one strand per pair. The titin strands/connections visualized with Tit1‐5‐H1.1 (Figure 3B) appear reasonably evenly stained along the length of the strand, and the staining of kinetochore fibers also was more or less even and not punctate. The titin strands (tethers) visualized with T5460 appear punctate along the interzonal titin strands, and the kinetochore spindle fibers also appeared punctate (Figure 3C).

**FIGURE 4:**
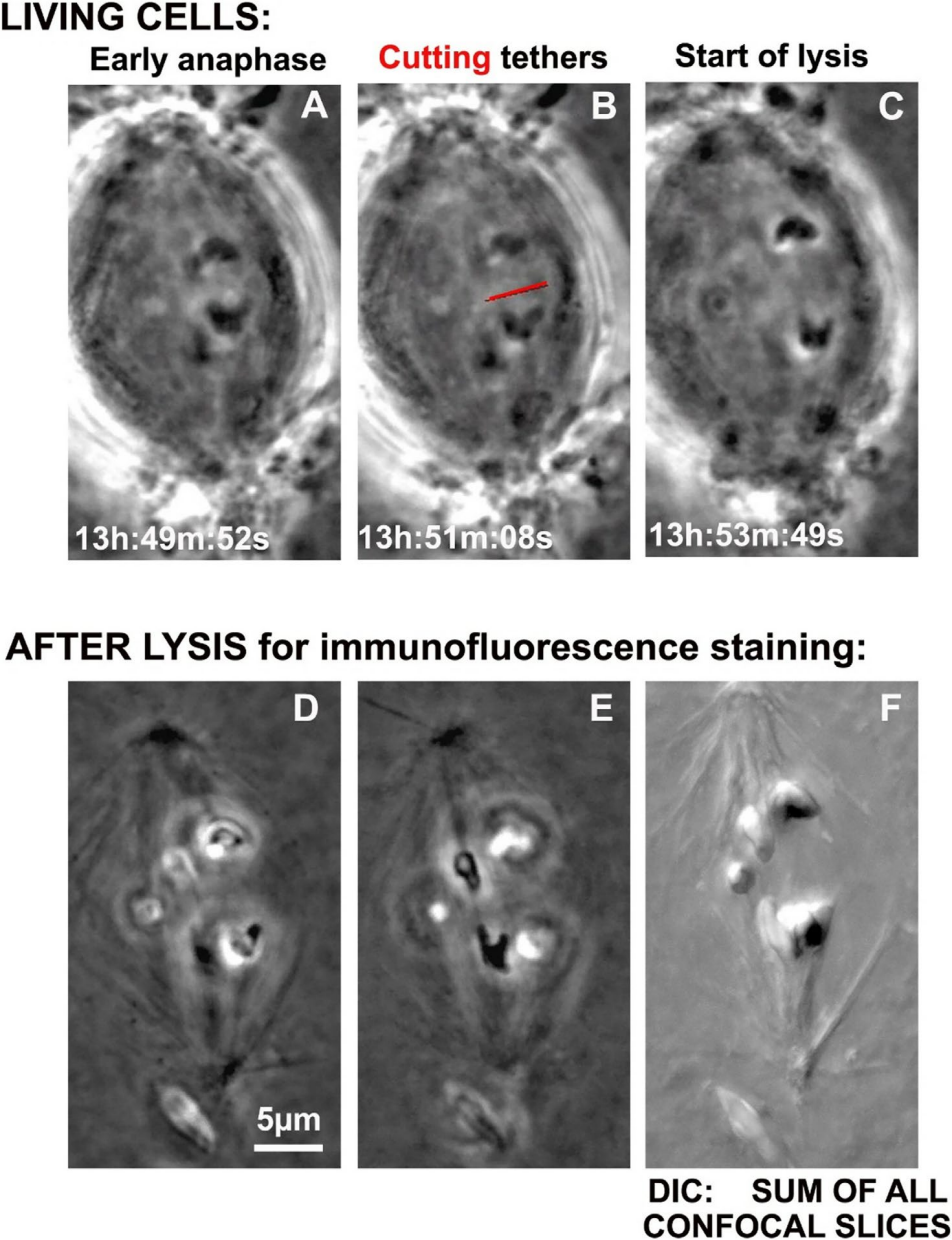
Illustrates a living crane‐fly spermatocyte (top row, A–C) in which the tethers of one separating pair were cut with a laser (red line in B). [The laser irradiation was performed as in Sheykhani et al. 2017, as one of the experiments done at that time.] This cell subsequently was stained with antibodies against titin (shown in Figures 5, 6, 7). As seen in the time stamps on the phase‐contrast microscope images of the living cell (A–C), anaphase movement continued after the irradiation until the cells were lysed in preparation for immunofluorescence. Figure illustrate the cytoskeleton of the cell after lysis, showing that the chromosomes keep their original positions, and illustrating that only the cytoskeleton (the spindle) remains after lysis in cytoskeleton stabilizing buffer: The rest of the cell is washed away. The DIC image (F) is of the same cell after fixation and staining, as seen using the confocal microscope.

**FIGURE 5:**
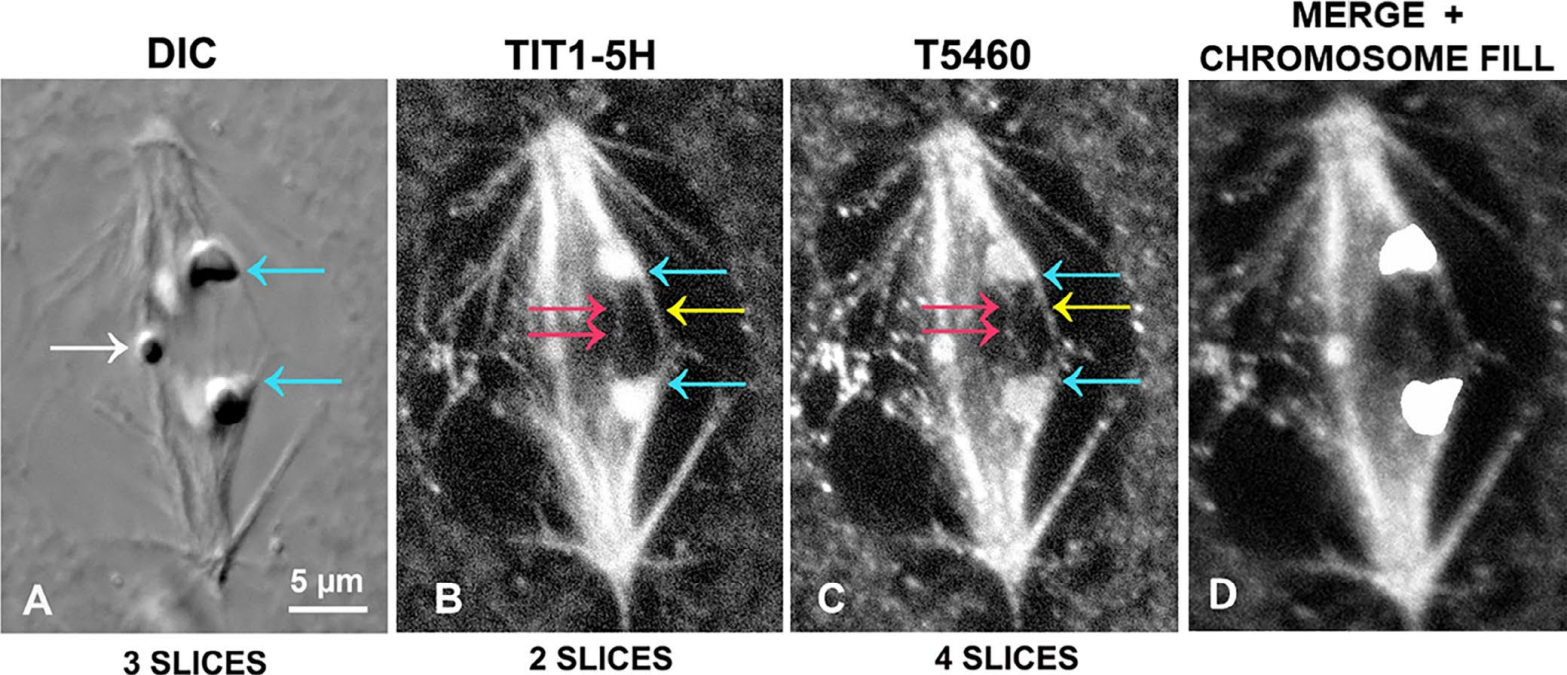
Illustrates the mid‐anaphase cell shown in Figure 4 after staining with anti‐titin antibodies Tit1‐5H‐1.1 and T5460. Figure is in the focal plane of the cut tethers. The three pairs of separating half‐bivalents were in three different focal planes, which are presented separately. The white arrow in the DIC image (A) points to a sex chromosome at the equator. Blue arrows point to the two half‐bivalents that had their tethers cut with the laser microbeam. The red arrows point to a tether (titin strand) that appears severed—it is joined to the top telomere but not to the bottom telomere. The yellow arrow points to the other titin strand associated with the same chromosome pair: It is straight as it extends from the top telomere but it is at an angle to the bottom telomere, and doesn't seem to connect to the bottom telomere. These varying tether‐telomere connections are highlighted in the merged and chromosome‐filled (D).

**FIGURE 6:**
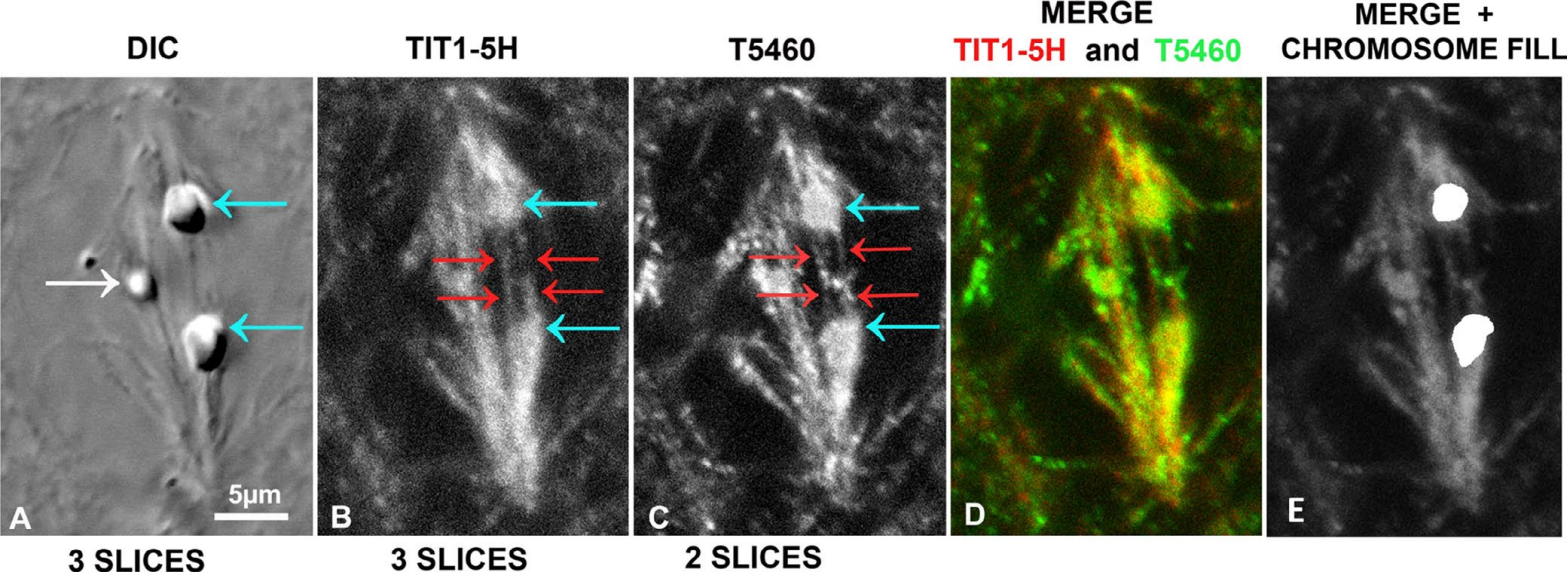
Illustrates the same mid‐anaphase spermatocyte shown in Figures 4 and 5, stained with anti‐titin antibodies Tit1‐5H‐1.1 and T5460, in a focal plane slightly displaced in the Z‐axis from that in Figure 5, illustrating a different pair of half‐bivalents. The white arrow in the DIC image points to an in‐focus sex chromosome at the equator (the same sex chromosome that was slightly out of focus in Figure 5). Blue arrows point to the two separating half‐bivalents that have titin‐strands connecting their two telomeres. The red arrows point to the two titin strands, stained by the antibodies listed above the images. The 3D merged image shows that each of the two strands is stained by the two different anti‐titin antibodies, Tit1‐5H1.1 (colourised red) and T5460 (colourised green). Sex chromosome kinetochore fibers are strongly stained as are the autosomal kinetochore fibers; other spindle regions are less strongly stained. (E) Is a merged image with in‐focus chromosomes filled in white.

**FIGURE 7:**
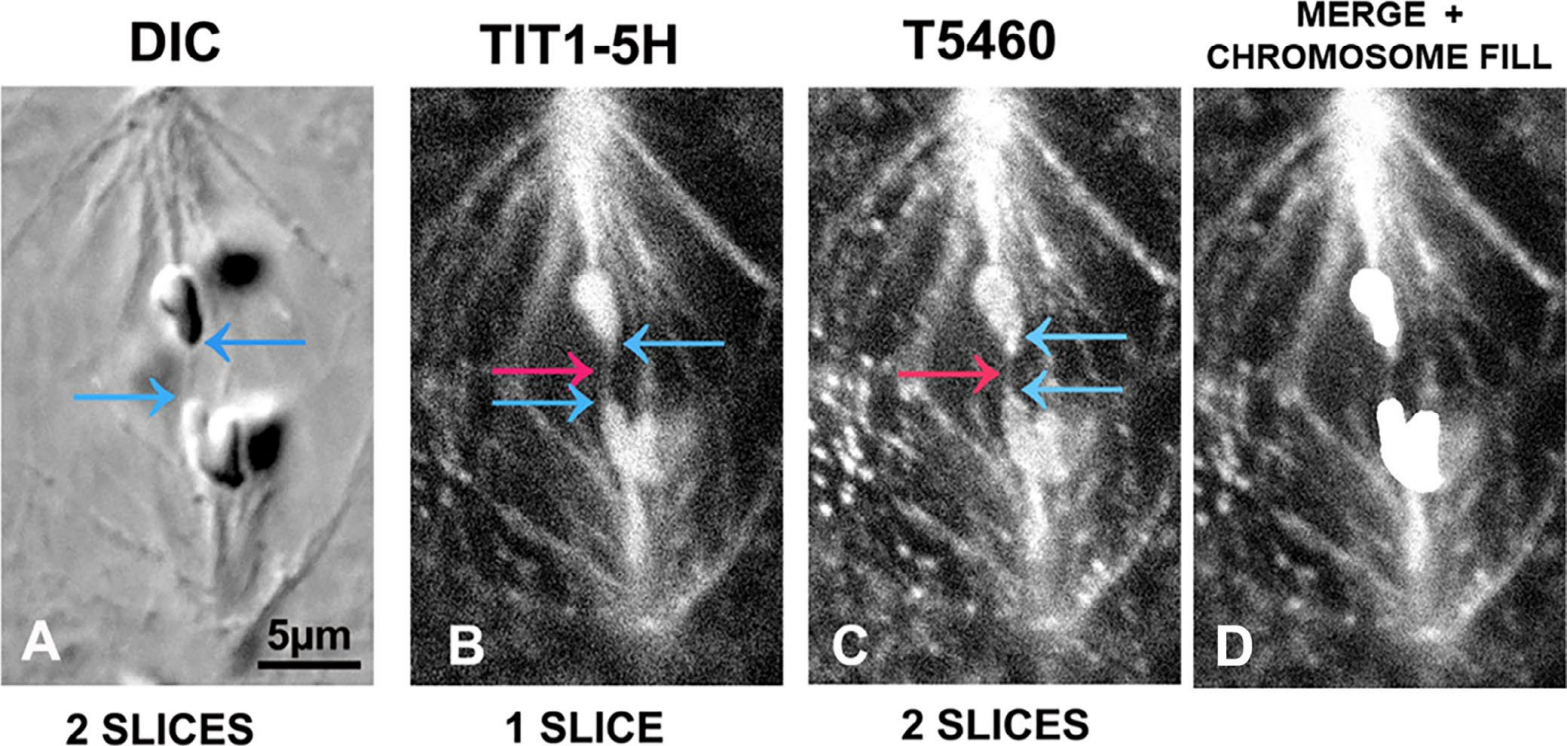
Illustrates the same mid‐anaphase cell shown in Figures 5 and 6, stained with anti‐titin antibodies Tit1‐5H‐1.1 and T5460. The images in figure are in a different focal plane than the others, showing the third pair of separating half‐bivalents. Blue arrows point to the separating half‐bivalents and red arrows point to the one titin strand connecting the telomeres that we were able to identify. As in an earlier section of this cell, spindle poles are strongly stained, the autosomal kinetochore fibers are strongly stained, but other spindle regions are less strongly stained.

Titin strands were studied in a cell in which tethers were cut with a laser microbeam (as described in Sheykhani et al. 2017): the two laser‐irradiated strands were disconnected from one of the two telomeres while the not‐irradiated strands were connected to both partner telomeres, as illustrated in Figures 4, 5, 6, 7. The in vivo irradiation prior to lysis for immunofluorescence is shown in phase‐contrast microscopy in early anaphase (Figure 4A), at the time the laser irradiated the tethers between one separating pair of chromosomes (red line in Figure 4B), and at the time that lysis buffer was added and the cell was beginning to lyse (Figure 4C). As reported previously (Sheykhani et al. 2017), each chromosome continued normal movement after irradiation of the tethers. In the cell in Figure 4, chromosome movements continued during the 2½ min between irradiation and lysis (cf. chromosome positions in Figure 4B,C). Phase‐contrast microscope images of the cell after lysis are shown in Figure 4D to illustrate the correspondence both with living cell images and with the DIC image of the cell in the final immunofluorescence preparation (Figure 4F). The images also illustrate that adding the lysis buffer prior to fixation preserves only the cytoskeleton (the spindle): the rest of the cell washed away in the lysis buffer. In this cell we identified titin strands connecting each of the three separating pairs of half‐bivalents.

The titin strands associated with the irradiated tethers are seen in (Figure 5B–D), in fluorescence images stained with both Tit1‐5 H1.1 and T5460. The *left* titin strand (red arrows in Figure 5B,C) seems “disorganized”: it does not extend in a straight line from its connection at the top telomere, and it seems to be disconnected from the bottom telomere. The *right* titin strand (Figure 5B,C, yellow arrow) seems attached to the top telomere; from there, it extends in a straight line at an angle to (not toward) the bottom telomere (bottom blue arrow) and seems to terminate in a disorganized “bundle” of titin. Thus, as expected from cutting tethers in live cells (Sheykhani et al. 2017; Forer et al. 2018), the laser seems to have severed both titin strands that connect the partner chromosomes.

In the same cell, the titin strands associated with the not‐irradiated tethers connect the partner telomeres. Two titin strands associated with one autosome pair are in a slightly different plane of focus than the irradiation (cf. 5A and 6A). This chromosome pair is in the same lateral position as the irradiated chromosome pair but is in a focus plane 2 to 3 confocal slices (each ~0.35 μm) away from the irradiated tethers. The separating chromosomes (blue arrows), the titin strands (red arrows), and one sex chromosome (white arrow) are visible in this plane of focus. Each of the two trailing arms of the separating pair of chromosomes is connected by a titin strand. [The rightmost titin strand almost seems to have an associated “bow‐tie”, seen in the *T5460* image (Figure 6C) and seen in green in the merged image (Figure 6D), perhaps due to slight damage from out‐of‐focus laser irradiation.] We could identify only one titin strand in the third (and last) pair of separating autosomes in this cell (Figure 7B–D). The chromosome arms that are connected are pointed to by blue arrows (cf. the phase‐contrast image in Figure 4E) and the titin strand (tether) by a red arrow. Thus, we have identified titin strands in all three pairs of separating half‐bivalents; the irradiation disconnected the titin strand in one pair of chromosomes, while the other two pairs of partners remained connected by titin strands (tethers).

In sum, we regularly have observed titin strands connecting separating anaphase chromosomes after staining early and mid‐anaphase spermatocytes with anti‐titin antibodies TIT1‐5‐H1.1 and T5460, and we have shown that the titin strands are damaged by laser microbeam irradiation, as are tethers (Sheykhani et al. 2017). If the titin strands we identified indeed represent tethers, we expect similar strands to be present in later stages of anaphase. Since tethers are present until late anaphase, we expect that all chromosome pairs have titin strands since all have tethers, and we expect titin strands to connect separating chromatids in meiosis‐II spermatocytes since we know that tethers connect meiosis‐II anaphase chromosomes. We now present cells that demonstrate these points.

**FIGURE 8:**
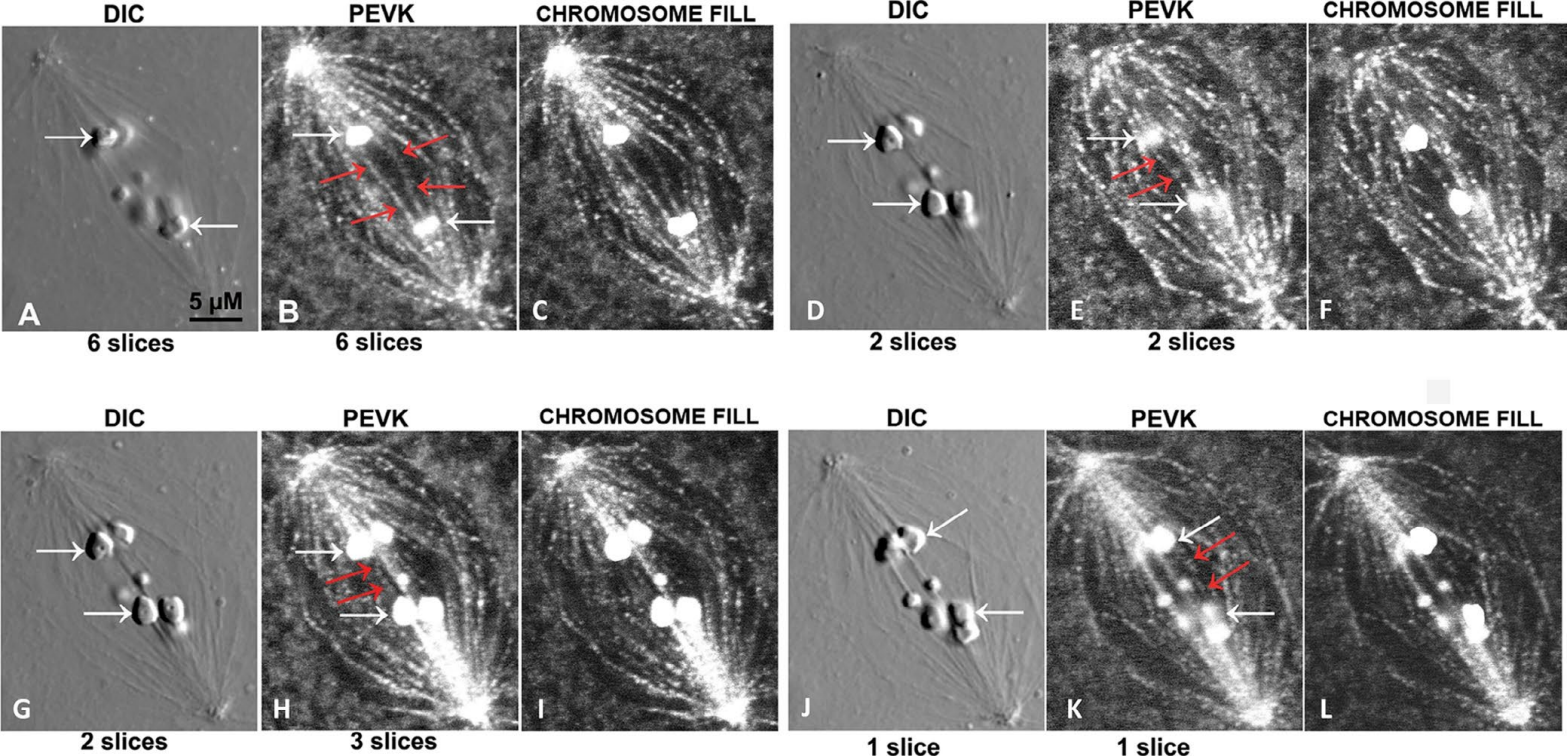
Illustrates with both DIC images and fluorescence images one mid‐anaphase spermatocyte stained with anti‐titin antibody PEVK. The images are in 4 different focal planes, showing that 1 or 2 titin strands connect each of the 3 separating pairs of half‐bivalents: Two per pair (shown in B and C); two per pair (shown in D–I); and one for the pair in (K) and (L). This shows that each separating pair is connected by tethers. White arrows in each image point to two separating half bivalents, red arrows point to the titin strands associated with the indicated half bivalents. In addition to tethers, sex chromosome and autosomal kinetochore fibers are strongly stained, spindle poles are strongly stained, and long titin strands outside the spindle extend along the cell long‐axis, apparently in the cell cortex.

All chromosome pairs are connected by titin strands in single cells, as illustrated in Figure 8, a mid‐to‐late anaphase cell stained with PEVK antibody. The titin strands are pointed to by red arrows and the associated chromosomes by white arrows. The chromosome pair pointed to in A has two connecting titin strands. The chromosome pair pointed to in C and E, in two slightly different planes of focus, has two connecting titin strands (pointed to in D and F). The chromosome pair pointed to in G has only one connecting titin strand visible, probably because the second strand was obscured by the stained kinetochore fiber of the nearby sex chromosome. Thus, all pairs are connected, two with two titin strands and the third with only one strand that we could identify.

**FIGURE 9:**
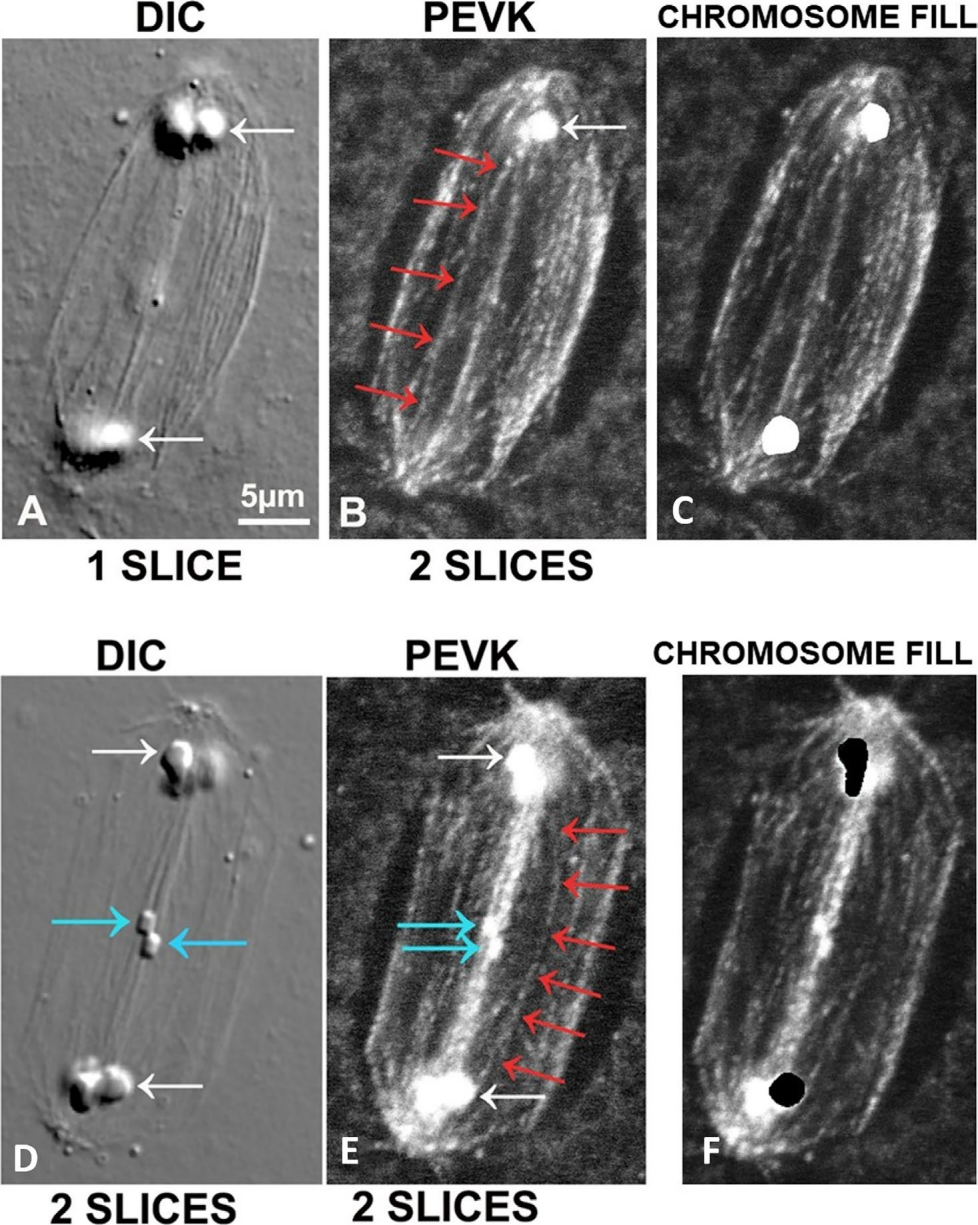
Illustrates two different planes of focus in one late‐anaphase spermatocyte stained with anti‐titin antibody PEVK. Blue arrows in (D, E) point to the two sex chromosomes that have started to move to opposite poles. White arrows point to the separated half‐bivalents, and these chromosomes are filled in (C) (white) and (F) (black). In (B), the bottom half‐bivalents are not in focus in the plane of the long titin strand. The red arrows point to the titin strands (tethers) that extend between the separated half bivalents. In addition to staining of tethers, the sex chromosome kinetochore fibers are stained strongly, though unevenly. Long titin strands extend in the pole‐to‐pole direction outside the spindle area. The sex chromosomes and autosomes are strongly stained.

Titin strands connect telomeres in late anaphase cells also, as shown in a cell stained with antibody PEVK (Figure 9), that illustrates two pairs of partner autosomes connected by titin strands. The chromosome pairs are pointed to by white arrows, the titin strands by red arrows, and the two sex chromosomes (that have started their anaphase segregation to the poles) are pointed to by blue arrows. These data identify titin strands in late anaphase connecting telomeres that are around 20 μm apart.

Figures 8 and 9 illustrate titin strands in two necessary conditions if the titin strands indeed represent mitotic tethers: (1) that tethers connect all chromosome pairs in anaphase and (2) that tethers are present in late anaphase. We now present data illustrating titin strands connecting separating chromatids in meiosis‐II spermatocytes.

**FIGURE 10:**
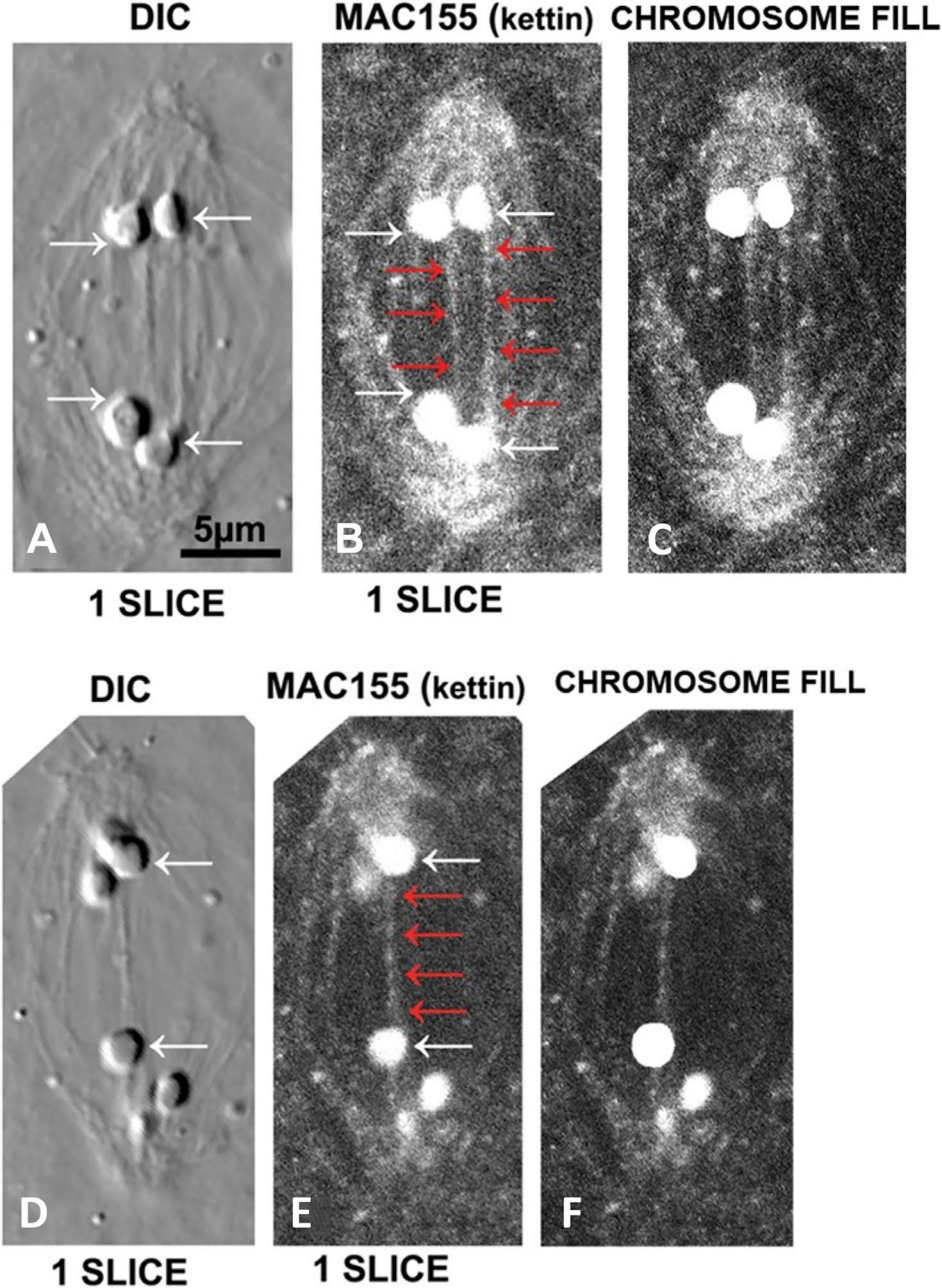
Illustrates two different late anaphase meiosis‐2 spermatocytes stained with the anti‐titin (anti‐kettin) antibody MAC155. In meiosis‐2, four chromatids move to each pole. We have identified single titin strands between 3 of the separating pairs of chromatids, two in one cell and one in the other, pointed to by the red arrows in (B, E). White arrows point to the separating chromatids and red arrows point to the associated titin strands. In addition to staining tethers, MAC155 strongly stained the chromosomes. Indicated chromosome pairs are also filled (C, F).

Titin strands connect separating chromatids in secondary spermatocytes, as illustrated in two cells stained with antibody MAC155, in Figure 10, in which white arrows point to the chromatids and red arrows to connecting titin strands. Figure 10A–C illustrate two separating pairs of chromosomes in a late‐anaphase cell, with each pair connected by one titin strand. Figure 10D–F illustrate one pair of chromatids in another cell, with partner chromatids connected by one titin strand. The titin strands clearly connect separating partner chromosomes, and unlike meiosis‐1, only one tether connects partner meiosis‐2 chromosomes.

In summary, using four different anti‐titin antibodies, we have identified titin strands that extend between (and connect) partner telomeres on chromosomes moving to opposite poles during anaphase in both meiosis‐I and meiosis‐II crane‐fly spermatocytes. We identified titin strands between separating autosomes in all stages of anaphase, early to late, for multiple chromosomes per cell. Most likely, they are present in all separating pairs. We identified one or two strands for each separating chromosome pair in primary spermatocytes and one for each separating chromosome pair in secondary spermatocytes. We never saw more than two strands connecting partner chromosomes in meiosis‐I, or more than one in meiosis‐II. And we never saw strands between chromosomes that were not partners. These descriptions of titin strands match the requirements of mitotic tethers deduced from laser irradiation experiments.

## 4 Discussion

A major finding of our experiments is that titin‐containing strands extend between separating telomeres during anaphase in both meiosis‐I and meiosis‐II crane‐fly spermatocytes, connecting the telomeres. We identified these titin strands using four different anti‐titin antibodies, each of which binds to a different part of titin. If we include the three different antibodies used by Fabian, Troscianczuk, and Forer (2007); Fabian, Xia, et al. (2007), that bind to three separate regions of titin different from the ones we used (Figure 1), there are *seven* different antibodies to titin that stained titin strands that connect partner telomeres. While it is possible that any given antibody binds off‐target to something other than titin, it seems unlikely in the extreme that seven different antibodies, binding to seven different parts of titin, all bind off‐target to the same single cytoskeletal component that extends between telomeres. Furthermore, the spindles we stained were cytoskeletons. Because we lysed the cells before fixation using a cytoskeleton‐stabilizing buffer, there were no components present other than cytoskeletal spindle components. Therefore, our data indicate that titin strands are actual components of the spindle cytoskeleton, including (but not limited to) the mitotic elastic tether system defined as elastic using laser microbeam irradiations to cut chromosome arms in anaphase (LaFountain et al. 2002; Forer et al. 2017) and defined morphologically using electron microscope tomography (Forer and Otsuka 2023). While we cannot offer definitive proof that titin strands are components of tethers, our observations of titin strands satisfy the properties of tethers known from laser experiments (LaFountain et al. 2002; Forer et al. 2017), namely that in crane‐fly primary spermatocytes there are two tethers connecting each separating pair of chromosomes and not more than two; that in the second meiotic division, meiosis‐II, separating chromosomes are connected with one tether per separating pair, as in mitotic cells (Forer et al. 2017); that all chromosome pairs in each spermatocyte are connected by tethers; and that tethers (titin‐stained strands) are severed using laser microbeam irradiations (Sheykhani et al. 2017). That titin strands satisfy all these morphological expectations suggests that titin is a component of mitotic tethers. While it is possible to contend that the titin strands are part of other connections between separating telomeres, we do not know of any such bridges (connections) that fit the same criteria. Other “bridges” between separating chromosomes do exist, such as the well‐characterized ultrafine DNA bridges, but as discussed in detail elsewhere (e.g., Forer and Otsuka 2023), ultrafine DNA bridges are not present in each cell, though tethers are; when ultrafine DNA bridges *are* present there are too few of them per cell for them to be tethers; and ultrafine DNA bridges slow down poleward movement whereas tethers do not. We do not know of any other mitotic “bridges” or “connections” that have the same properties as the elastic tethers and as the titin strands that we see. Thus, we think that the titin strands are part of the mitotic tether system originally defined by laser‐cutting experiments.

### 4.1 Could Titin Be Responsible for Tether Elasticity?

Tethers are elastic and titin is a molecular spring intimately involved in the stretch and elasticity of skeletal and cardiac muscle, as discussed in the Introduction. Since titin molecules can produce force in the pico‐Newton range and higher, e.g., Erickson 1997, more than enough to move chromosome arm fragments backward, and since, like tethers, titin elasticity is altered by phosphorylation and dephosphorylation, as discussed in the Introduction, we suggest that titin functions as an elastic component in mitotic tethers. One test of this suggestion might be to stain the putative tethers with antibodies against phosphorylated titin, to see if the titin strands between telomeres are phosphorylated when they are short (say < 5 μm) but are not phosphorylated (or phosphorylated less strongly) when they are longer, as predicted by data on when tethers lose elasticity (Kite and Forer 2020; Forer et al. 2021).

That titin's elasticity can depend on its phosphorylation state also fits the suggestion that titin may be responsible for mitotic tether elasticity. PKC‐dependent phosphorylation of cardiac titin in the PEVK region increases the passive tension of titin, which is reversed (titin loses elasticity) after dephosphorylation in the same region by PP1 (Hidalgo et al. 2009); this is similar to the loss of mitotic‐tether elasticity caused by PP1. [Phosphorylation and dephosphorylation in the N2B region of cardiac titin have the opposite effect, however reviews: Ahmed and Lindsey (2009); Krüger and Linke (2011).] Further circumstantial evidence is that PP1 is active in mitotic cells during anaphase and is present near tethers, i.e., PP1 is associated with the chromosomes and is present in the region between separating anaphase chromosomes (Fernandez et al. 1992; Andreassen et al. 1998; Trinkle‐Mulcahy et al. 2003, 2006). These circumstantial evidences are consistent with titin causing tether elasticity.

In sum, our data on titin strands connecting separating telomeres in anaphase support the hypothesis that titin is a component of mitotic tethers that were characterized using laser microbeam experiments.

Another major finding is that titin is a component of spindles. All four antibodies stained various parts of mitotic spindles: chromosomes, kinetochore microtubule bundles, spindle area in general, spindle poles, as well as cell cortex and cleavage furrow, similar to the staining by three different anti‐titin antibodies used by Fabian, Troscianczuk, and Forer (2007); Fabian, Xia, et al. (2007). Using the same reasoning as above, it is extremely unlikely that these seven different antibodies, to different regions of the titin molecule, would all bind to the same off‐target sites. The crane‐fly spermatocyte locations that stained strongly with anti‐titin antibodies include kinetochore spindle fibers, cell cortex, and cleavage furrow; from this, it would seem that in spermatocytes titin is present in spindle regions that contain microtubules (the spindle proper) and that titin is present in regions that contain actin and myosin (cell cortex and cleavage furrows). Because the antibodies stained chromosomes, titin also might be a component of chromosomes.

Titin functions in non‐muscle cells have been considered before, and several articles have considered cytoplasmic elements we have pointed to in our studies. Some suggested titin is present in chromosomes. Houchmandzadeh and Dimitrov (1999) and Woodcock and Dimitrov (2001) suggest titin is important in chromosome structure, as did Machado and Andrew (2000). Others suggested roles in cell cortex activity. Toffali et al. (2023) describe multiple roles of different titin isoforms in lymphocytes, in activities such as cell stiffness and microvilli structure: in lymphocytes, titin isoforms are involved with the morphogenesis of plasma membrane microvilli and resilience to passive cell deformation. Others suggested that titin is involved with the cytoplasmic cytoskeleton: Eilertsen et al. (1994) and Cavnar et al. (2007) suggested roles for titin in stress fibers and epithelial cell brush borders. Maruyama et al. (1977) and Hashimoto et al. (1984) suggested titin is important in membrane/cell cortex structure. Hashimoto et al. (1984), Glassner et al. (1985), Pudles et al. (1990), Fabian, Xia, et al. (2007), Qi et al. (2008), and Toffali et al. (2023) suggested that titin is present in a cytoplasmic matrix/cytoskeleton. Zastrow et al. (2006) suggested that titin is involved in nuclear structure/organization via its association with lamins. Fabian, Xia, et al. (2007) pointed to titin staining of nuclear membranes and cleavage furrows. Overall, these previous articles suggested that cytoplasmic titin might interact with cytoskeletal components involved with the cell cortex and cytoplasmic cytoskeleton, presumably interacting with actin and myosin.

**FIGURE 11:**
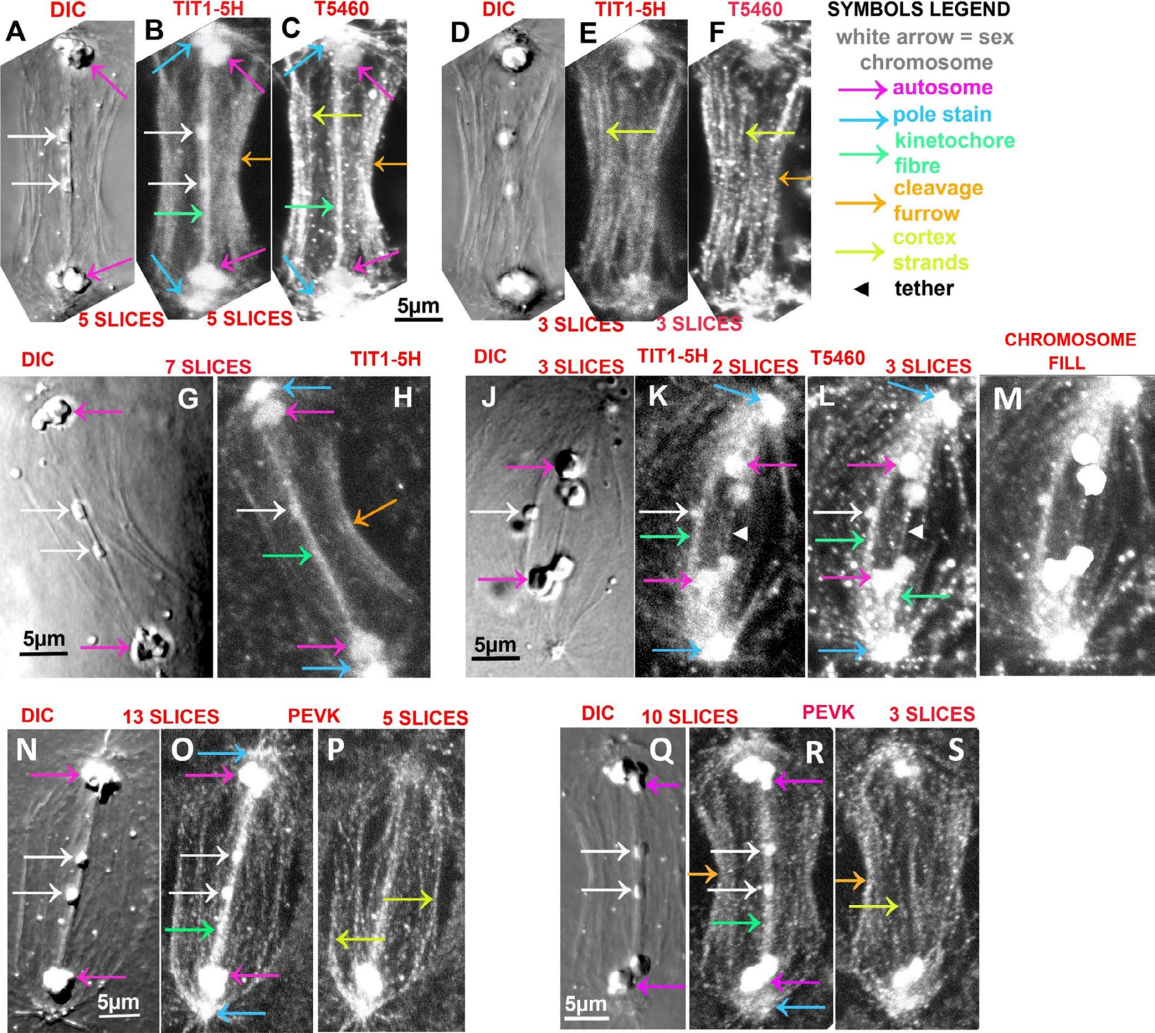
Illustrates staining of the spindle by anti‐titin antibodies. The antibodies used in specific images are labeled above each panel. The arrows point to objects indicated by the colours in the symbols legend. (A–F) Show one cell in late anaphase, seen (in A–C) in a mid‐spindle plane and seen (in D–F) in the cell cortex. The sex chromosomes (white arrows) are segregating to the poles. There is staining of spindle poles (blue arrows), of sex‐chromosomes (white arrows) and their kinetochore fibers (green arrows), of autosomes near the poles (red‐violet arrows), of titin strands in the cortex (yellow‐green arrows) and of the cleavage furrow (orange arrows). (G, H) (one cell), and (N–S) (two cells) illustrate staining of spindle poles (blue arrows), of autosomes (red‐violet arrows), of sex chromosomes (white arrows) and their kinetochore fibers (green arrows), of cleavage furrows (orange arrows), and of cortical titin strands (yellow arrows). (J–M) (one cell) illustrate staining of autosomes (red‐violet arrows), of a sex chromosome (white arrows), of kinetochore fibers of a sex chromosome and of an autosome (green arrows), of spindle poles (blue arrows), and of tethers (white arrowheads).

It should not be much of a surprise if titin functions within the cell cortex, cleavage furrow, and cytoplasmic cytoskeleton because in muscle cells titin is associated with and functions together with actin and myosin, and actin and myosin are associated with the cell cortex, cytoplasmic cytoskeletons, and cleavage furrows. It might be a surprise that titin is associated with (or is present in regions near to) spindle microtubules because microtubules generally are assumed to be the only cytoskeletal component in spindles; this unexpected conclusion might even lead one to suspect that the supposed association of titin with spindle microtubule regions might represent artefactual staining of microtubules rather than represent a functional or neighborly association. We think the titin staining of microtubule regions is *not* “artefactual” off‐target staining of spindle microtubules, and that the staining is indeed of titin. One reason is that in crane‐fly spermatocytes titin “co‐staining” with microtubules is seen with all seven different antibodies to titin, so it is unlikely to be due to off‐target staining. For another, in our studies (and those of Fabian, Xia, et al. 2007) there usually is not a one‐to‐one correspondence of titin staining with spindle microtubules: titin staining includes some apparently “co‐localized” staining of microtubules—especially in kinetochore microtubule bundles—but often titin stain co‐localizes with only parts of individual microtubule bundles even in the kinetochore fiber bundles (Figures 1, 3, 6, 8, 9, 11). That there is not always an exact one‐to‐one correspondence of titin and spindle microtubules speaks against titin artefactually (off‐target) staining spindle microtubules. Further, staining of spindle (microtubule regions) by anti‐titin antibodies has been reported in several other studies: Wernyj et al. (2001), Zastrow et al. (2006), Fabian, Troscianczuk, and Forer (2007); Fabian, Xia, et al. (2007), and Mikelsaar et al. (2010, 2012) have described titin staining of mitotic spindles in different cell types. Further, Mikelsaar et al. (2010, 2012) showed that titin is associated with centrioles of human, mouse, and zebrafish cells, similar to our strong staining of spindle poles (Figures 3, 5, 7, 8, 11). Finally, two of the antibodies used in the present article were formed against specific immunogen peptides: antibody Tit1 5H1.1 recognizes and reacts with the six‐peptide series *AVNKYG*, and antibody PEVK recognizes and reacts with the 12‐peptide series *CEVVLKSVLRKR*. Analysis using *BLAST* indicated that no significant stretches of either sequence are present in alpha‐tubulin or beta‐tubulin isomers in *Drosophila* (fruit fly) or *Culex* (mosquito). That means that the targets stained by these two antibodies are not present in spindle microtubules. Thus our data indicate that titin itself is present in spindles per se, as well as in tethers. We think the staining represents titin in the vicinity of spindle microtubules, not artefactual staining of microtubules, and we think that titin may be involved with one or more non‐microtubule cytoskeletal force systems involved in chromosome and other movements in spindles. We suggest that titin may associate with some kinetochore microtubules (or other spindle microtubules) in general and may function with them, or may function together with spindle myosin, actin, or spindle components other than microtubules that may be involved in chromosome movement. This would not be unique since it is quite common for multiple cytoskeletal force producers to work together (e.g., Rodriguez et al. 2003; Pimm and Henty‐Ridilla 2021; Dunkley et al. 2022). Indeed, various evidences point to spindle actin and myosin participating in chromosome movements in spindles (e.g., Forer and Pickett‐Heaps 1998; Forer et al. 2003; Weber et al. 2004; Pickett‐Heaps and Forer 2009; Woolner et al. 2008; Vilmos et al. 2009; Rump et al. 2011; Johansen et al. 2011; Sheykhani, Shirodkar, and Forer 2013; Sheykhani, Baker, et al. 2013; Mogessie and Schuh 2017; Burdyniuk et al. 2018; Fegaras and Forer 2018; Plessner et al. 2019; Fegaras‐Arch et al. 2020; Sugizakia et al. 2021; Yim et al. 2024), so it would not be surprising if spindle titin interacts with microtubules, actin, myosin and/or a spindle matrix (Johansen et al. 2011) to produce or regulate force in mitotic spindles. It even is possible that microtubules may be more involved in regulation of chromosome segregation via checkpoints, chromosome alignment, chromosome orientation, etc., than in producing the forces for chromosome movement (as suggested, for example, by Pickett‐Heaps and Forer 2009). We think that mitotic tethers and mitosis in general can be added to the list of titin functions that occur in non‐muscle cells.

A second “surprise” might be that at least 6 of the 7 anti‐titin antibodies stained chromosomes strongly and regularly, and thus that titin may be part of chromosome structure. Various anti‐titin antibodies have been reported to stain chromatin and chromosomes (e.g., Machado et al. 1998; Machado and Andrew 2000; Wernyj et al. 2001; Zastrow et al. 2006; Fabian, Xia, et al. 2007; Qi et al. 2008; Mikelsaar et al. 2010; Mikelsaar et al. 2012; Toffali et al. 2023; our data) and titin is associated with the nucleomatrix (review in Simon and Wilson 2011). Thus, there are some data that point to titin perhaps being a structural component of chromosomes. In addition to direct staining of chromosomes, one can argue that, in light of our hypothesis, titin *must* be a component of chromosomes because tethers contain titin and tethers arise from chromosomes. Likewise, the presence of titin in chromosomes corroborates our conclusion that tethers extend from chromosomes. Electron microscope images of presumed tethers between telomeres (Forer and Otsuka 2023) show that tethers appear between separating telomeres at the start of anaphase, extending from what appears to be chromatin, and they remain between the separating telomeres throughout anaphase. Thus, they arise from the telomere regions of chromosome arms and from the chromosomes themselves. That titin is present in chromosomes is reinforced by observations in various cells that show that titin antibodies stain chromatin and/or chromosomes (e.g., Machado et al. 1998; Wernyj et al. 2001; Zastrow et al. 2006; Fabian, Xia, et al. 2007; Qi et al. 2008; Mikelsaar et al. 2010, 2012; Toffali et al. 2023; this paper), and by experiments by Machado and Andrew (2000) that indicate that mutations in *Drosophila* titin alter chromosome structure as well as cause defects in muscle organization. Our evidence for titin being part of tethers, and thus part of chromosomes, supports the earlier experimental data that titin is involved with chromosome structure, and supports the suggestions from theoretical considerations of the physics of chromosome stretching that titin might have an important role in chromosome structure (Houchmandzadeh and Dimitrov 1999; Woodcock and Dimitrov 2001).

*In summary*, evidence from staining of titin with four different antibodies supports the hypothesis that titin strands (mitotic tethers) connect separating telomeres during anaphase, confirming the suggestion by Fabian, Xia, et al. (2007). Our data suggest that titin is part of the elastic mitotic tether system that connects separating chromosomes, and that titin may be responsible for at least some of the elasticity of the tethers. We suggest that titin functions in important ways in non‐muscle cells, including possible involvement with force production in mitotic spindles and in cell cortices and cleavage furrows.

## Author Contributions

A.F. and R.S. conceived and designed the study. D.E., R.S., M.J., and A.F. collected the data. D.E., A.F., and R.S. analyzed the results. D.E., A.F., and R.S. wrote the manuscript. M.K. and A.‐V.M. provided PEVK and Tit1 5H1.1 antibodies, respectively. A.F. supervised the study and secured funding. All authors revised and approved the manuscript.

## Disclosure

Dr. Forer received a grant from the Natural Science and Engineering research Council of Canada. NSERC played no role in the study design, data collection and analysis, decision to publish or preparation of the manuscript.

This submission is unpublished and not under consideration elsewhere. All submitted images are in compliance with Cytoskeleton's image manipulation policy.

## Conflicts of Interest

The authors declare no conflicts of interest.

## Data Availability

The data that support the findings of this study are available from the corresponding author upon reasonable request.
